# Optimizing electrode structure of carbon nanotube gas sensors for sensitivity improvement based on electric field enhancement effect of fractal geometry

**DOI:** 10.1038/s41598-021-96239-1

**Published:** 2021-08-17

**Authors:** Taicong Yang

**Affiliations:** School of Microelectronics and Communication Engineering, Chongqing University, Chongqing, 400044 China

**Keywords:** Electrical and electronic engineering, Mechanical engineering, Electronic and spintronic devices

## Abstract

With the rapid development of carbon nanotubes gas sensor, the sensitivity of the sensing response is becoming more and more demanding. Different from the traditional studies on gas-sensitive materials, this paper combines the microscopic dimensional effects and physical properties of fractal geometry theory from the structure and morphology of sensor devices. The electrode structures of carbon nanotubes gas sensor is designed and optimized by Hilbert–Piano curve. Simulation experiments demonstrate that the electric field intensity and hot spot distribution of the fractal electrode are superior to those of the traditional interdigital electrode. Moreover, a novel chemiresistive gas sensor is fabricated combining the characteristics of carbon nanotubes and fractal geometry, and a test with exposure to nitric oxide showed that the sensors with fractal electrode structures improved the gas sensing sensitivity over sensors with traditional geometrical structures. It provides a new idea for the exploration of gas sensing technology.

## Introduction

Bionic olfactory, also known as electronic nose, is an electronic system composed of gas sensors and pattern recognition algorithms^1^. It can achieve portable, effective non-invasive detection, and is often used in medical diagnosis, food manufacturing, industrial and agricultural production, aerospace, and other fields^2–5^. Pattern recognition algorithm includes feature extraction and pattern analysis. As the low threshold and open database, it has attracted the attention of many researchers and its technology is relatively mature^6,7^. In contrast, the sensor technology includes the research of gas sensitive material, the design of sensing element, the manufacturing technology and so on, which involves physics, mathematics, chemistry, materials science, technology, statistics and a variety of modern science and technology^8–12^. So, the performance of gas sensors has become the key technology restricting the development of electronic nose systems^13,14^.

Conventional gas sensors, such as semiconductor-based metal oxide sensors and polymer-based chemical resistance sensors, etc., the response principle is usually that the gas-sensitive material undergoes an oxidation-reduction reaction with gas molecules, and the changing electrical signals, such as resistance, current, and voltage, are induced and conducted through a transducer or measuring electrode to perform qualitative or quantitative measurement of the gas^15,16^. For metal oxide semiconductor sensors, although they can react with many target gases, these sensors operate at high temperatures, typically above 200 °C^17,18^. Conductive polymer sensors, on the other hand, are less sensitive and selective, although they can respond at lower temperatures^19^.

With the recent interest in nanomaterials, carbon nanotubes (CNTs) are expected to be better gas-sensitive materials because of their large specific surface area, high number of absorption points and their reactivity at room temperature^20–25^. In 2000, Kong et al. discovered that CNTs exhibits the electrical properties of a p-type semiconductor and used this as a theoretical basis for the preparation of a CNTs gas sensor, which is gas sensitive to NO_2_ and NH_3_ at room temperature^23^. In 2014, Daewoong et al. used a novel fabrication technique to make a hydrogen sensor using thin sheets of multi-walled carbon nanotubes (MWCNTs) and evaluated its output performance^26^. However, carbon nanotubes also have disadvantages such as low sensitivity and long response time, thus further improving the performance of carbon nanotube sensors has become a hot topic of current research^27^.

Currently, most studies have been carried out to improve the overall gas-sensitive properties of CNTs by means of modification or compounding. For example, CNTs are modified and compounded by metal nanoparticles^28^, metal oxides^29^ and conducting polymers^30^. But, there are also problems of complex operation and poor stability^31^. However, little research has been done on the optimization of sensor structure and shape, including the design of the transducer structure and the improvement of the appearance of the sensor. Since increasing the intensity of the electric field between the electrodes can increase the carrier migration speed between the gas-sensitive material and the gas molecules, increase the oxidation–reduction reaction rate, and thereby improve the sensitivity of the sensor.

Fractal geometry is a kind of non-Euclidean geometry and was first proposed in 1975 by Benoit Mondelbrot, whose main characteristic is self-similarity, that is, complex geometric structure can be generated by simple iterative method^32^. This self-similar structure has three advantages when applied to gas sensors: large specific surface area, locally enhanced electric field and good repeatability.

In this paper, fractal geometry is combined with the structural design of MWCNTs gas sensor, and a simulation experiment is carried out to verify the effect of electric field intensity enhancement firstly. Moreover, we designed and manufactured a chemical resistance sensor with fractal structure electrode, and proved the performance of this sensor is better than the traditional interdigital electrode structure sensor through gas experiment. We aim to test the feasibility of multi-walled carbon nanotubes gas sensor structural optimization for performance improvement and the potential of fractal geometry in the development of gas sensors.

## Results

### Simulation results of electric field intensity of electrodes with different structures

**Figure 1:**
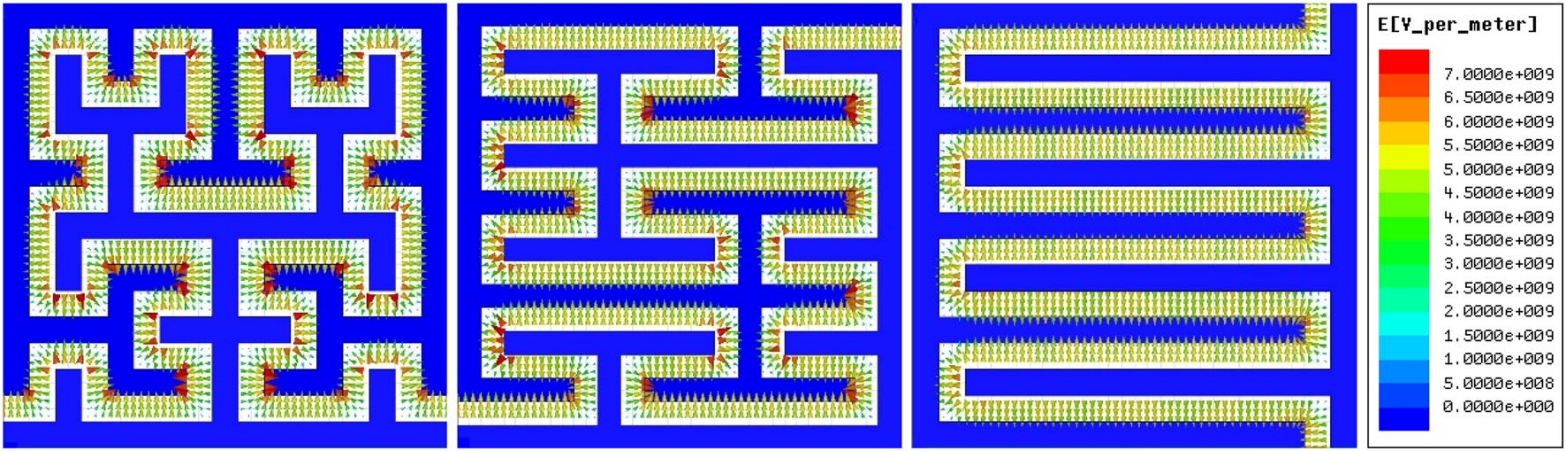
Simulation results of electric field intensity between sensor electrodes: (**a**) Hilbert–Piano curve a; (**b**) Hilbert–Piano curve b; (**c**) Interdigitated electrodes.

The results of Maxwell software electric field experiments are shown in Fig. 1 and Table 1. As can be seen from those, when the same voltage is applied to both metal plates, the electric field at the corners of the fractal electrode will be larger than that of the interdigital electrode, which can be called “hot spots”. And as the complexity of the curve increases, so does the number of hot spots and the intensity of the electric field. (The maximum electric field intensity of the fractal electrode is about 1.6 times that of the interdigital electrode, and there are more hot spots in curve a than in curve b).

**Table 1:** Maximum electric field intensity between electrodes.

Electric field intensity	E (V/m)
Hilbert–Piano curve a	9.81 × 10^9^
Hilbert–Piano curve b	7.87 × 10^9^
Interdigital electrode	6.17 × 10^9^

### Gas sensing results

**Figure 2:**
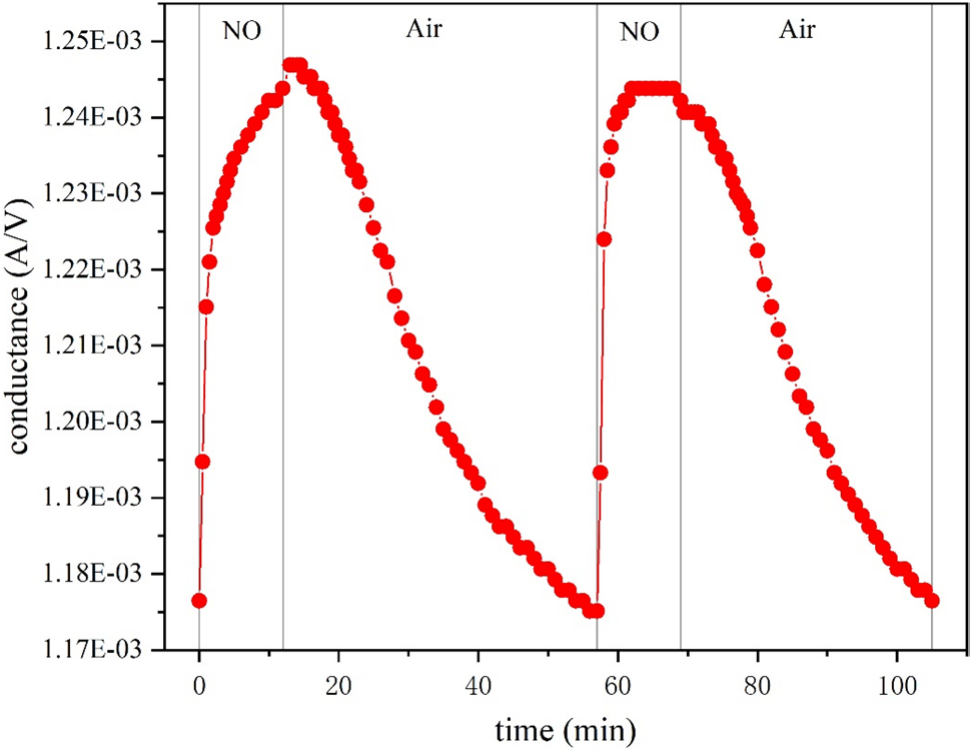
Conductance of MWCNTs to 100 ppm NO.

Gas sensing experiments of MWCNTs were carried out in a 325 ml gas chamber with 100 ppm nitric oxide (NO) while monitoring the resistance of the MWCNTs. As shown in Fig. 2, we observed that the conductance of MWCNTs can be substantially increased by exposure to NO. Simultaneously, two consecutive experiments with MWCNTs showed good reproducibility.

Based on the results of the simulation experiment, we chose Hilbert–Piano curve *a* as the electrode structure of the two fractal sensors for NO gas experiment, and compare them with the two traditional interdigital electrode sensors.

**Figure 3:**
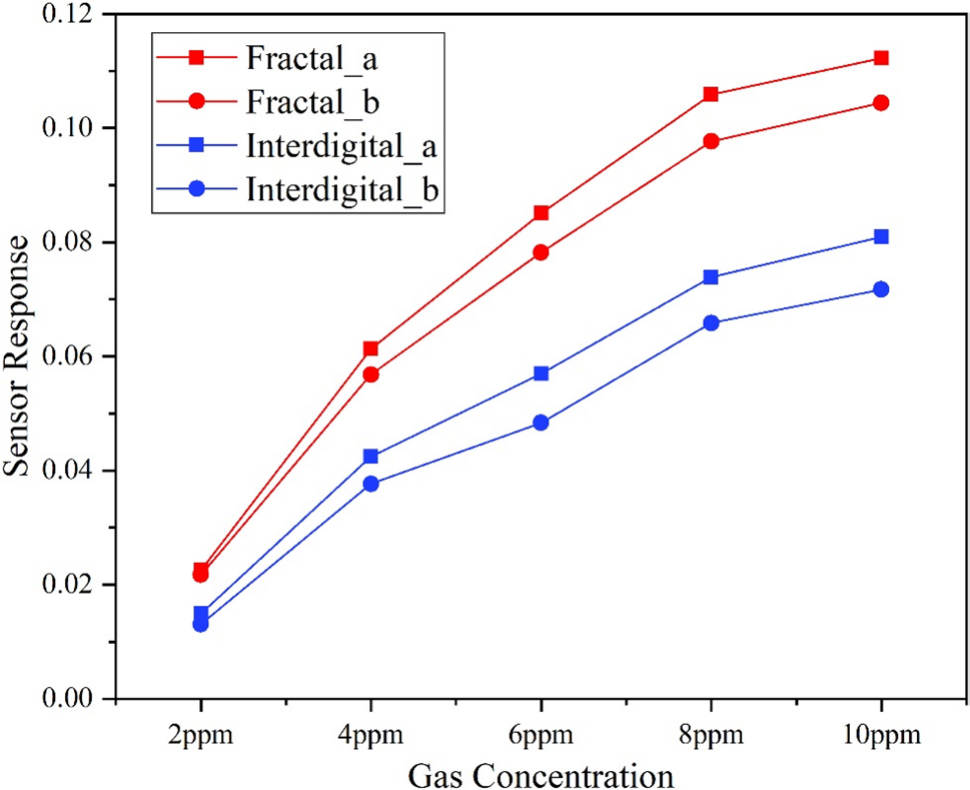
Curves of fractal and interdigital sensors responses to NO at concentration gradient.

The gas sensing results in sensitivity of the four sensors with NO concentration are shown in Fig. 3 below. The sensors have a good response in the low concentration of 2–10 ppm firstly. And the gas response follows the traditional power law model secondly. Finally, the response amplitude of the fractal electrode sensors were greater than that of the interdigital electrode sensors, with a minimum increase of 29% and a maximum of 76%. Moreover, the power consumption of the sensors were between 0.001 and 0.003 W as the resistances of the sensors varies between 2000 and 4000 ohms and the voltages across them varies between 2 and 4 V.

## Discussion

MWCNTs is p-type semiconductors with a large number of hole carriers at room temperature^23^. When it is exposed to NO, this results in the nanotube valence band being closer to the Fermi energy level, which allows the migration and enrichment of hole carriers in the nanotube, increasing the conductance of the nanotube.

From Fig. 5, it can be seen that when the side length of the Hilbert–Piano curves are reduced by a factor of 2 or 3, the number of new forms are 4 or 9 times the original number, and therefore the fractal dimension of the Hilbert–Piano curve:

1 $$D = \frac{\lg 4}{{\lg 2}} = \frac{\lg 9}{{\lg 3}} = 2$$

This suggests that Hilbert–Piano curves are far more complex than conventional Euclidean geometric curves (of dimension 1) and can even fill the entire plane (with a theoretically infinite number of orders). As a result, Hilbert–Piano curves can form a large number of corners or sharp angles. As the Gauss's law of electric field:

2 $$\begin{gathered} \oint_{S} {\overrightarrow {E} \cdot d\overrightarrow {S} } = \frac{\sigma \Delta S}{{\varepsilon_{0} }} \hfill \\ E\Delta S = \frac{\sigma \Delta S}{{\varepsilon_{0} }} \hfill \\ E = \frac{\sigma }{{\varepsilon_{0} }} \hfill \\ \end{gathered}$$

**Figure 4:**
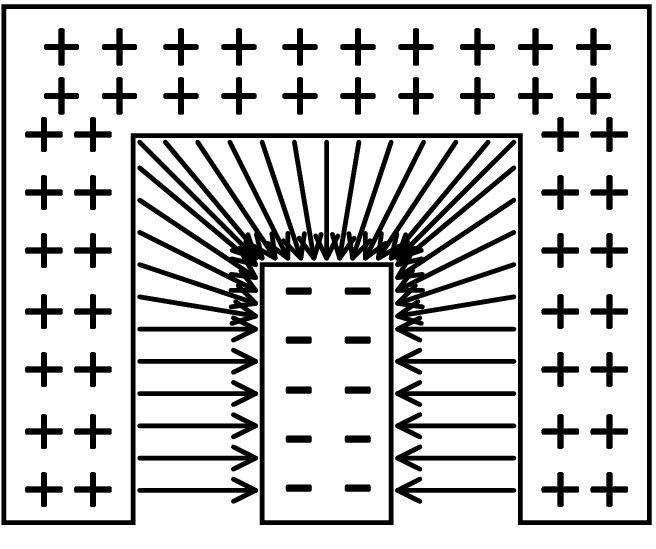
Schematic diagram of electric field distribution at different positions of electrodes.

The magnitude of the electric field intensity at a surface is proportional to the surface density of charge at that surface. So there is a high density of charge distributed on metal structures with sharp corners or large curvature profiles, creating a strong surface electric field. For the electrode structure of the sensor, the electric field line density at the vertices or corners of the electrodes is much greater than at the parallel electrodes, as shown in Fig. 4. Therefore, the more complex the electrode structure is, the more corners there are, and the more hot spots with large electric field intensity. From the differential form of Ohm’s law:

3 $${\mathbf{j}} = \sigma {\mathbf{E}}$$

It is known that the current density is proportional to the electric field intensity in the same direction. And drift speed of carriers under electric field force is:

4 $$\upsilon_{d} = \mu |E|$$

Therefore, the fractal structure can increase the electric field intensity between the electrodes inside the sensor, which in turn not only makes the electron transfer or totalization process easier to complete, but also increases the current density between the conductors, ultimately improving the sensitivity of the sensor.

## Methods

### The generation of fractal structure and electric field intensity simulation

The grammar composition algorithm is an algorithm that imitates the grammar generation method in linguistics to construct graphics. When studying the evolution and structure of plant morphology, American biologist Aristid Lindenmayer proposed a grammatical description method in 1986: Graftal, which later developed into an important branch of formal languages, called LS (L-System)^33^. In 1984, Smith first introduced LS to the field of computer graphics^34^.

LS grammar is a unique type of iterative process whose core concept is rewriting. As a formal language, LS grammar uses alphabets and symbol strings to express the initial form of the generated object, called Axiom, and then according to a set of production rewriting rules, each character in the initial form is sequentially replaced with a new character form, This process repeatedly replaces and rewrites, and finally generates the ultimate graphics.

**Figure 5:**
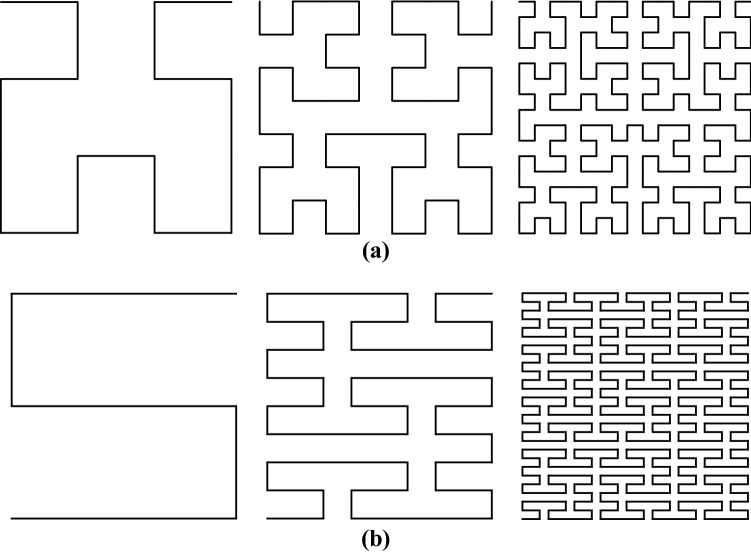
The first three orders of two Hilbert–Piano curves: (**a**) Hilbert–Piano curve a; (**b**) Hilbert–Piano curve b.

On a two-dimensional plane, the drawing rules of the LS grammar alphabet are as follows:

- F: Take one step in the current direction and draw a line;
- +: Rotate counterclockwise by δ°;
−: Rotate clockwise by δ°;

The alphabet is V, the initial Axiom is ω, and the generation rule is P. Following Eqs. (5) and (6), we generated two Hilbert–Piano curves (named curve a, curve b, and curve c) using two letters in the alphabet as double rules, respectively. The first three stages are shown in Fig. 5, respectively.

5 $$\left\{ {\begin{array}{*{20}l} {\delta = 90^\circ } \hfill \\ {\omega {:}\quad {\text{X}}} \hfill \\ {P_{1} {:}\quad X \to - {\text{YF}} + {\text{XFX}} + {\text{FY}} - } \hfill \\ {P_{2} {:}\quad Y \to + {\text{XF}} - {\text{YFY}} - {\text{FX}} + } \hfill \\ \end{array} } \right.$$

6 $$\left\{ {\begin{array}{*{20}l} {\delta = 90^\circ } \hfill \\ {\omega {:}\quad {\text{X}}} \hfill \\ {P_{1} {:}\quad X \to {\text{XFYFX}} + {\text{F}} + {\text{YFXFY}} - {\text{F}} - {\text{XFYFX}}} \hfill \\ {P_{2} {:}\quad {\text{Y}} \to {\text{YFXFY}} - {\text{F}} - {\text{XFYFX}} + {\text{F}} + {\text{YFXFY}}} \hfill \\ \end{array} } \right.$$

where “X” and “Y” only participate in string replacement and only draw on “F”.

We used these two fractal curves to design the electrode shape of the sensor. Because of the self-similar characteristics of fractal geometry, the physical properties of similar structures are also the same, so we intercepted the first two levels of the Hilbert–Piano curve for structural design, and interdigital electrodes of the same size were used for comparison. Electrostatic fields between the electrodes were simulated by the Maxwell software (ANSYS, America) in the Cartesian coordinate system based on Maxwell’s equations and finite element method.

### Sensor manufacture and gas sensing experiment

Because of the results of the simulation experiment, we chose Hilbert–Piano curve *a* as the fractal electrode structure. We also selected a copper clad plate as substrate as it is insulated from the outside, and has good adhesion to the electrodes, simultaneously. As for the gas sensitive material, the carboxylated multi-walled carbon nanotubes, produced by Chengdu Organic Chemicals Co., Ltd. (Chengdu, China China), Chinese Academy of Sciences was selected.

**Figure 6:**
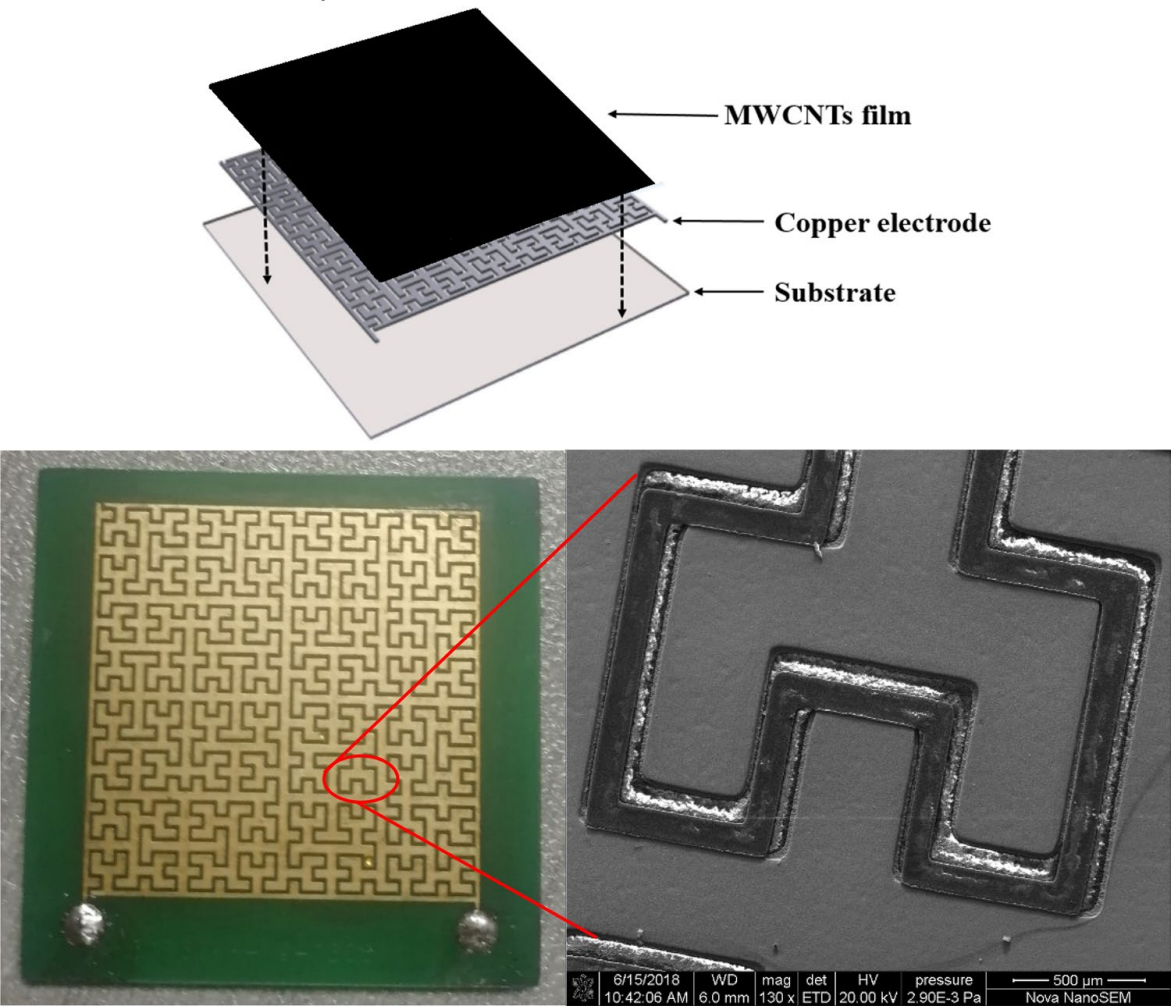
Top: schematic of the sensor. Bottom: appearance view of the sensor (left) and SEM micrograph of the fractal electrode (right).

The classical three-layer design of substrate-electrode-gas sensitive film has been chosen for the structure of the sensor^35–38^, the schematic of the sensor is shown in Fig. 6. We used microelectromechanical system (MEMS) processing to fabricate the sensor electrode. The copper layer, with a specified thickness, was first electroplated onto the substrate, then etched into the electrode structure by a chemical reaction method. Finally, a thin layer of gold was deposited onto the surface of the electrode by a gold deposition process (pre-treatment, nickel deposition, gold deposition and post-treatment) to increase conductivity and prevent oxidation. The aspect ratio and effective areas of the fractal electrode and the interdigital electrode was 1:1, wherein the width of the plate is 400 μm, the pitch is 200 μm, and the thickness is 30 μm.

We prepared translucent conductive carbon nanotube films by a combination of drop coating and spin coating with optimized process parameters. The carbon nanotubes were first dispersed by a combination of dispersant (TNWDIS, Carbon Nanotube Water Dispersant, Chengdu Organic Chemical Co., Ltd., Chinese Academy of Sciences) and deionized water, followed by ultrasonic treatment. This was performed by centrifugation to remove undispersed particles. The electrode plates were cleaned with ethanol and washed with deionized water prior to deposition. A small amount of dispersed CNTs was then dropped onto the electrode and the gas-sensitive film was prepared by spin coating using a Spin Coater (Institute of Microelectronics of the Chinese Academy of Sciences, China) at 800 r/min. The number of coats was controlled to obtain the required thickness and the resistance of the film was continuously monitored.

**Figure 7:**
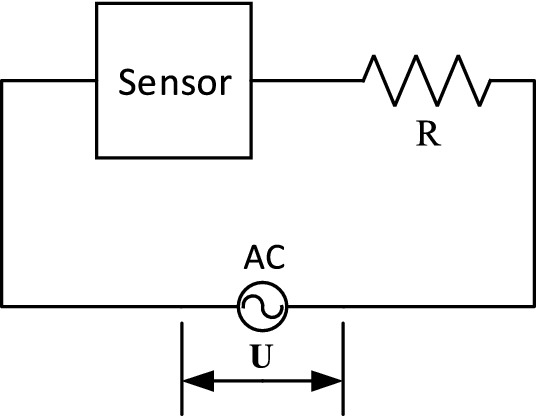
Experimental circuit.

It was connected in series with a fixed resistor to measure the response of the gas sensor, as shown in Fig. 7. An AC (alternating current) sine wave signal with an amplitude of 5V generated by VirtualBench (National Instruments, USA) was used as the input signal and the voltage *V* across the sensor was detected by a data acquisition card (DAQ, ART Technology, China). The impedance characteristics of the sensor were proportional to the input voltage *V* sensor. Thus, if we define the impedance of the sensor as *Z* and the voltage of the signal source as *U*, we have:

7 $$V = \frac{Z}{R + Z}U$$

**Figure 8:**
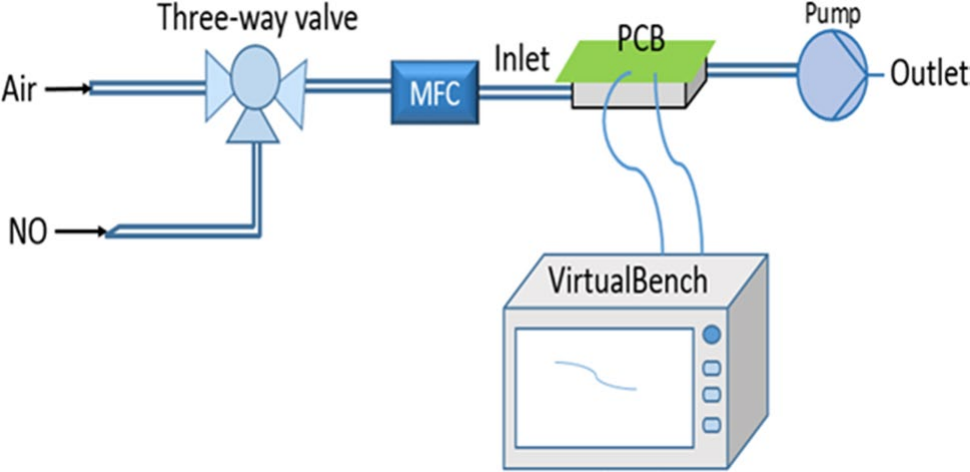
Gas sensing test system.

After absorption of the gas, the impedance of the sensor changed and the voltage changed across the sensor in the circuit was measured to calculate the corresponding gas concentration. In the test, the sensors were exposed to dry clean air for 3 min and then injected with the target gas (in this case nitrous oxide) for 3 min. Different concentrations of NO gas from 2 to 10 ppm were used in the test. The gas chamber is a 325 ml stainless steel box coated with PTFE. The sensors were soldered to a printed circuit board (PCB) and placed upside down in the box. A mass flow controller (MFC) was used to control the gas flow rate. All gas sensing experiments were carried out at room temperature because of the properties of carbon nanotubes. The test system is shown in Fig. 8.

The sensor response is defined as:

8 $$Sensor \, Response = \frac{{V - V_{0} }}{{V_{0} }}$$

where *V* is the sensor response at current concentration, *V*_*0*_ is sensor response at 0 ppm of stimulus

## Conclusions

Based on the previous work of the carbon nanotubes gas sensor, using multi-walled carbon nanotubes as a gas-sensitive material, this paper further investigates the microscopic size effect and physical properties of fractal geometry, and combines them with the electrode structure of gas-sensitive sensors to verify the advanced and feasibility of sensitivity enhancement of fractal geometry in carbon nanotubes gas sensing technology through simulation test and gas experiments with NO. It was showed that the design method can effectively improve the sensitivity of gas sensor, providing a new idea and method for improving the performance of carbon nanotube gas sensor. More importantly, the self-similarity property of fractal geometry can generate complex and regular physical structures by a simple iterative algorithm, which ensures the repeatability and stability of the carbon nanotubes gas sensing response.

In future work, fractal geometry and gas sensing technology can be co-designed and integrated for fabrication. MEMS and CMOS technologies are used to further reduce the size of sensor pieces^39^, integrate multiple sensor pieces to form a sensing array, and integrate data processing modules and artificial intelligence algorithms to achieve chip-level package manufacturing and improve the comprehensive performance of the electronic nose system.
